# Long-Term Structural and Functional Restoration of the Retina After Inverted Internal Limiting Membrane Flap Surgery for Idiopathic Full-Thickness Macular Holes

**DOI:** 10.3390/diagnostics16183017

**Published:** 2026-09-17

**Authors:** Oskar Lorenc, Krzysztof Safranow, Anna Machalińska

**Affiliations:** 1 First Department of Ophthalmology, Pomeranian Medical University, 70-204 Szczecin, Poland; oskar.lorenc@pum.edu.pl; 2 Department of Biochemistry and Medical Chemistry, Pomeranian Medical University, 70-204 Szczecin, Poland

**Keywords:** optical coherence tomography (OCT), macular hole surgery, inverted ILM flap, glial proliferation, microperimetry, multifocal electroretinography

## Abstract

**Objectives**: To characterise longitudinal structural and functional retinal restoration after inverted internal limiting membrane (ILM) flap surgery for idiopathic full-thickness macular holes (FTMHs), with a particular emphasis on postoperative glial proliferation, outer retinal restoration, and structure–function relationships. **Methods**: This retrospective consecutive case series included 46 eyes of 44 patients with idiopathic FTMH treated using the inverted ILM flap technique. Spectral-domain optical coherence tomography (SD-OCT), best-corrected visual acuity (BCVA), microperimetry, fixation stability, and multifocal electroretinography (mfERG) were performed 1, 6, and 12 months postoperatively. **Results**: Anatomical closure was achieved in all eyes. Complete restoration of both the ELM and EZ increased from 8.7% at 1 month to 45.7% at 6 months and 54.3% at 12 months, whereas severe glial proliferation decreased from 30.4% to 15.2% at 12 months. BCVA, retinal sensitivity, fixation stability, and central mfERG responses improved predominantly between 1 and 6 months postoperatively. In multivariable analysis, lower closure-pattern grade (B = −0.073, 95% CI: −0.141 to −0.005; *p* = 0.035) and lower glial proliferation grade (B = −0.091, 95% CI: −0.155 to −0.027; *p* = 0.006) were independently associated with better BCVA at final visit. **Conclusions**: Retinal healing after inverted ILM flap surgery is a coordinated, time-dependent process involving regression of postoperative gliosis, restoration of the outer retinal microstructure, and multimodal functional recovery. Longitudinal integration of OCT with microperimetry, fixation analysis, and mfERG provides a comprehensive framework for understanding postoperative retinal remodelling beyond anatomical hole closure.

## 1. Introduction

Although the inverted internal limiting membrane (ILM) flap technique has markedly improved anatomical closure rates in full-thickness macular holes (FTMHs), postoperative visual recovery remains highly variable, even among eyes with successful anatomical closure [1,2]. This discrepancy indicates that successful closure of the macular hole represents only the first stage of retinal healing, whereas subsequent structural and functional restoration represents the ongoing process that determines the final visual outcome [2]. Therefore, understanding the biological pathways underlying postoperative retinal remodelling has become increasingly important.

Optical coherence tomography (OCT) has substantially advanced the evaluation of postoperative retinal healing by enabling detailed assessment of the foveal microstructure. Restoration of the external limiting membrane (ELM) and ellipsoid zone (EZ) has consistently been associated with improved postoperative visual acuity, whereas persistent disruption of the outer retinal layers may limit functional recovery despite successful anatomical closure [3,4,5]. However, structural restoration assessed by OCT alone does not fully explain the variability of postoperative visual function, suggesting that additional mechanisms contribute to retinal repair.

One such mechanism may be postoperative glial remodelling. Previous studies suggested that Müller cells activation and proliferation may contribute to the healing response following macular hole surgery [6]. During the early postoperative period, glial tissue fills the foveal defect and has been proposed to provide a structural scaffold that could facilitate retinal tissue reapproximation and subsequent restoration of the outer retinal microstructure [7,8]. Nevertheless, postoperative gliosis appears to play a dual role. While an appropriate glial response may support retinal repair, excessive or persistent glial proliferation may interfere with physiological retinal remodelling and photoreceptor alignment, thereby limiting functional visual recovery despite successful anatomical closure [5,9,10].

Although individual aspects of postoperative macular recovery have been extensively investigated [8,11,12], retinal healing after inverted ILM flap surgery is a dynamic process involving both structural and functional changes over time. The temporal sequence of outer retinal reconstruction, regression of postoperative glial proliferation, and recovery of retinal function is not fully understood. Furthermore, previous studies have provided important insights into specific components of postoperative retinal restoration, including outer retinal reconstruction, microperimetric outcomes, mfERG changes, and postoperative glial proliferation [12,13,14,15]. However, these parameters have largely been investigated separately, while their concomitant longitudinal assessment within a single multimodal evaluation has been less extensively explored.

Therefore, the aim of the present study was to characterise the longitudinal dynamics of morphological and functional retinal restoration during a 12-month follow-up period after inverted ILM flap surgery for FTMH. Particular attention was directed toward postoperative glial proliferation patterns, the restoration of the outer retinal layers, and their associations with visual acuity, retinal sensitivity, fixation stability, and electrophysiological recovery.

## 2. Materials and Methods

### 2.1. Study Design

This retrospective, consecutive case series included 44 patients (46 eyes) who underwent surgery for idiopathic FTMHs using the inverted flap technique at the 1st Department of Ophthalmology, Pomeranian Medical University in Szczecin, Poland. The study included consecutive patients who were treated between April 2022 and October 2024. Written informed consent for surgical treatment was obtained from all participants. The study was conducted in accordance with institutional guidelines and the tenets of the Declaration of Helsinki. The Bioethical Commission of Pomeranian Medical University reviewed the study protocol and determined that formal ethical approval was not required due to the retrospective analysis of anonymised patient data.

The inclusion criteria included the presence of an idiopathic FTMH confirmed by spectral-domain (SD) OCT and the availability of complete postoperative follow-up examinations at 1, 6, and 12 months. The exclusion criteria included high myopia, diabetic retinopathy, age-related macular degeneration, and previous vitreoretinal surgery or glaucoma.

### 2.2. Surgical Procedure

All procedures were performed by a single experienced vitreoretinal surgeon (A.M.) using a standardised 25-gauge pars plana vitrectomy technique with an inverted ILM flap. All included eyes were pseudophakic at the time of surgery. Following core vitrectomy, posterior vitreous detachment (PVD) was induced when not already present, with triamcinolone acetate used for enhanced vitreous visualisation. After staining with brilliant blue G (BBG), the ILM was circumferentially peeled around the macular hole while preserving a residual flap attached to the hole margin. The flap was subsequently inverted to cover the foveal defect. Fluid–air exchange followed by tamponade with 20% sulfur hexafluoride (SF6) gas. Patients were instructed to maintain a prone position for 3 days postoperatively, in accordance with the institutional postoperative protocol.

### 2.3. OCT Imaging and Morphological Analysis

Postoperative retinal morphology was assessed using SD-OCT performed with the Heidelberg Spectralis OCT system (Heidelberg Engineering, Heidelberg, Germany). OCT acquisition was conducted simultaneously with infrared reflectance imaging using a 30° scanning field that corresponded to approximately 8.8 mm of retinal coverage. All the examinations were obtained in high-speed acquisition mode with enhanced depth imaging (EDI-OCT). To improve image quality and reduce speckle noise, the Automatic Real-Time (ART) function was applied during image acquisition, with each displayed B-scan representing the average of 20 repeated frames acquired at the same retinal location. Only scans with a quality score ≥ 25 were included in the analysis. Quantitative and qualitative OCT analyses were performed using the integrated Heidelberg software calliper tool (Heidelberg Eye Explorer, version 1.12.1.0; Heidelberg Engineering, Heidelberg, Germany). The entire OCT volume scan was reviewed for morphological assessment, with grading based on cross-sectional B-scans passing through the central foveal region and closest to the anatomical centre of the macular hole. Baseline FTMH size was additionally categorized according to the CLOSE Study Group classification based on minimum linear diameter (MLD) [16].

**Figure 1:**
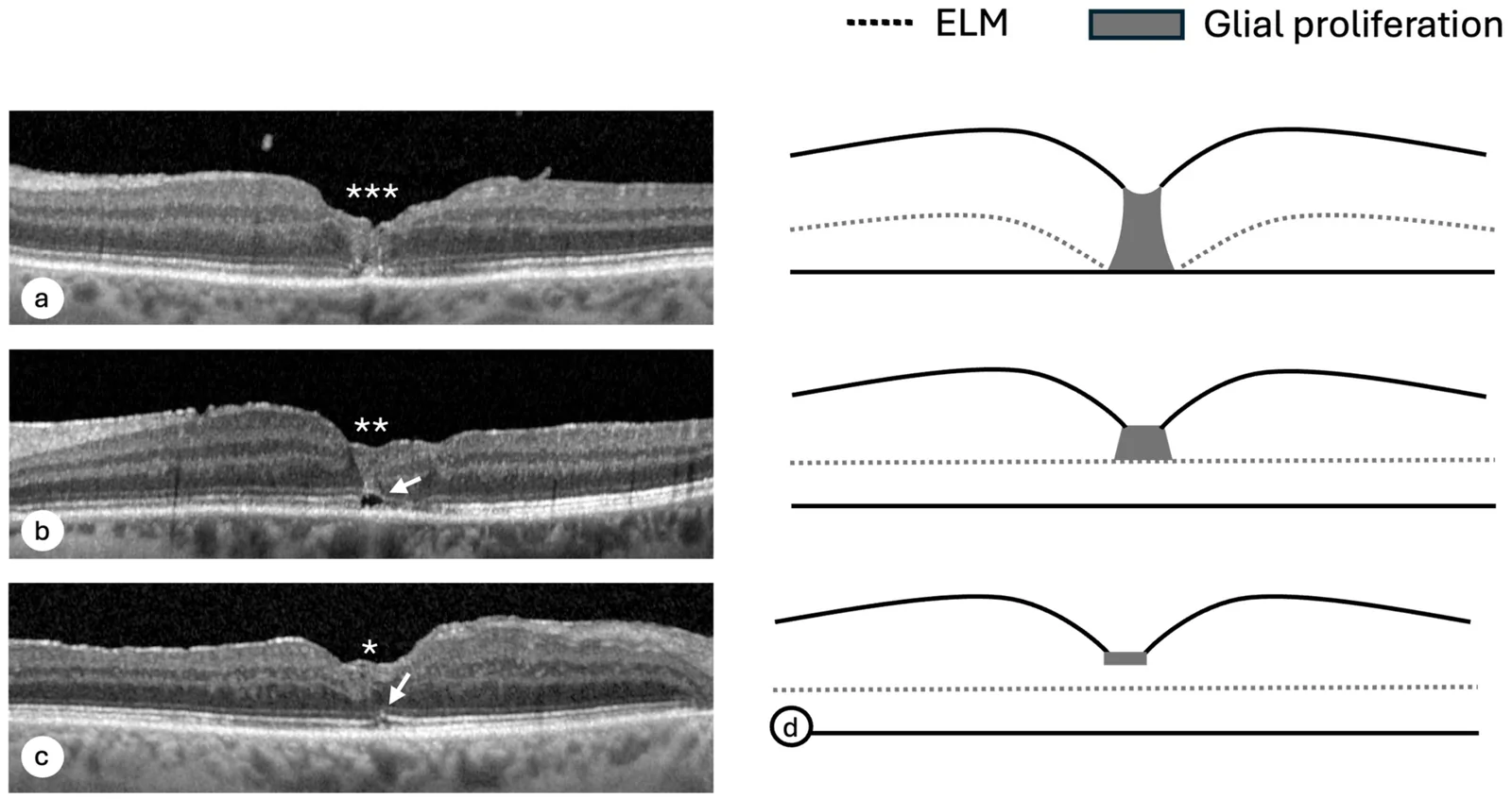
Representative OCT images demonstrating the three types of glial proliferation. (**a**)—In grade 3 glial proliferation, the moderately reflective lesion replaces the entire intraretinal layer (asterisks). (**b**)—A lesion with moderate reflectivity (asterisks) in grade 2 proliferation is located at and above the external limiting membrane (ELM; arrow). (**c**)—In grade 1 proliferation, the hyperreflective lesion (asterisk) is observed as superficial proliferation situated above the ELM (arrow). (**d**)—Schematic representation of three grades of glial proliferation.

The postoperative closure phenotype was assessed according to the integrity of the outer retinal layers and the presence and extent of glial proliferation. Glial proliferation was identified as a moderately reflective lesion located within the central fovea and classified according to a previously described grading system [10] (Figure 1). Grade 3 was defined as glial proliferation extending throughout the entire intraretinal thickness, grade 2 as proliferation located at and above the ELM, grade 1 as superficial proliferation situated above the ELM, and grade 0 as the absence of glial proliferation.

**Figure 2:**
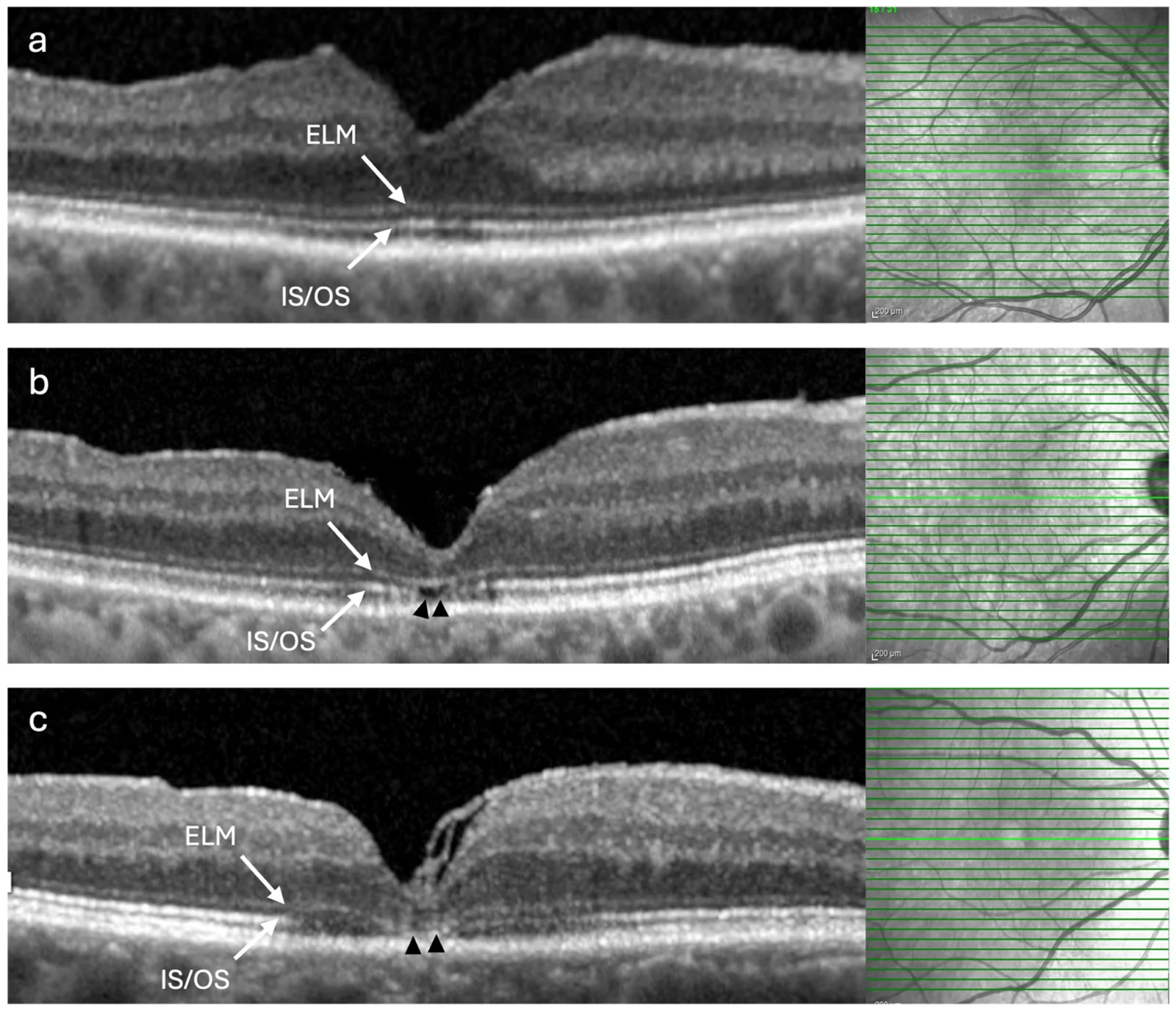
Representative OCT images obtained after MH repair, demonstrating the closure patterns of the photoreceptor IS/OS junction and ELM at the fovea. (**a**)—Type 1—restoration of both the ELM and the photoreceptor IS/OS junction. (**b**)—Type 2—disruption of the IS/OS junction (black arrow) but a restored ELM. (**c**)—Type 3—disrupted photoreceptor IS/OS junction and the ELM (black arrow).

Closure pattern classification was based on the integrity of the outer retinal layers, as proposed by Wakabayashi et al. [17]. Specifically, the restoration pattern was categorised according to the integrity of the EZ (previously referred to as the IS/OS junction) and ELM signals. Type 1 represented complete restoration of both the EZ and the ELM, type 2 indicated a disrupted EZ with preserved ELM integrity, and type 3 represented disruption of both the EZ and the ELM (Figure 2).

Morphological grading was performed by an experienced vitreoretinal surgeon (AM). In cases of uncertainty, the OCT images were additionally reviewed by a second specialist (OL) to achieve consensus classification. For interobserver reproducibility assessment, all OCT images were subsequently independently reassessed by the second specialist, who was blinded to the initial grading, functional outcomes, and postoperative time point.

### 2.4. Functional Assessment

The examinations included measurements of best-corrected visual acuity (BCVA) via both Snellen and ETDRS charts using a modified ETDRS chart containing 40 letters distributed across eight rows, with five letters per row and a logarithmic progression of letter size (0.1 logMAR between consecutive rows), covering a visual acuity range from 0.2 to 1.0. BCVA was recorded as the total number of correctly identified letters. For statistical analyses, the obtained scores were converted to standard ETDRS-equivalent letter scores by adding 45 letters to each result. Letter-by-letter scoring (0.02 logMAR per letter) was used in accordance with the original ETDRS protocol described previously [18,19].

Microperimetry and mfERG examinations were performed using the same standardised protocols routinely implemented in our department and described previously by our group [20]. Microperimetry was conducted with the MAIA microperimeter (CenterVue, Padova, Italy) using a 37-stimulus grid centred on the fovea and the 4–2 threshold strategy. The analysis included the mean retinal sensitivity, fixation stability parameters (P1 and P2), and bivariate contour ellipse area (BCEA) indices.

Multifocal electroretinography (mfERG) was performed using the RetiScan System (version 1021.3.0.0; Roland Consult, Brandenburg an der Havel, Germany) in accordance with the International Society for Clinical Electrophysiology of Vision (ISCEV) standards. Pharmacological mydriasis was achieved with 10% phenylephrine hydrochloride, and refractive correction was adjusted to a viewing distance of 0.3 m. Monocular stimulation employed a black-and-white array comprising 103 hexagons (distortion factor 4), extending 30° from the central fixation point to the edge of the stimulus field. Stimulus luminance was 100 cd/m^2^ and Michelson contrast was 97%. DTL fibre electrodes were used for signal acquisition. Six recordings were obtained from each eye and subsequently averaged. The acquisition settings included a 10–300 Hz bandpass, with the notch filter disabled, and an artefact-rejection threshold of 8% for an amplifier range of ±100 μV. Post-acquisition processing included automatic double smoothing and reduction in line interference, followed by manual adjustment of cursor position when required. All examinations were conducted by the same trained orthoptist. Post-acquisition mfERG analysis was performed by a single trained ophthalmologist who was aware that the recordings were obtained from eyes after FTMH surgery but was masked to the postoperative time point, OCT findings, and other functional assessments. Although the primary mfERG analysis focused on R1 because FTMH primarily affects the central foveal region, P1 response density and implicit time were also evaluated in rings R2–R6 as secondary regional measures.

### 2.5. Statistical Methods

All eyes were treated as independent observations in the statistical analyses. Two patients contributed both eyes, and no adjustment for within-subject correlation was applied. The normality of quantitative variables was assessed using the Shapiro–Wilk test. Since the distributions of most quantitative variables were significantly different from a normal distribution, non-parametric tests were used: the Wilcoxon signed-rank test for comparisons of measurements performed at different time points and the Spearman rank correlation coefficient test for correlations between measurements. These tests were also used for rank variables, e.g., glial proliferation grade. Dichotomous variables were compared between time points with McNemar’s test. Additionally, exploratory multivariable linear regression analyses were performed separately for each postoperative time point to assess the independent associations of glial proliferation and closure pattern with functional outcomes. Both morphological parameters were included simultaneously in each model, together with age, minimum linear hole diameter, and the corresponding preoperative functional value as covariates. Associations for which *p* was <0.05 were considered to be statistically significant. Given the exploratory nature of the structure–function analyses, no formal adjustment for multiple comparisons was applied, and the corresponding *p*-values should be interpreted as exploratory. Interobserver agreement was assessed using weighted Cohen’s kappa.

## 3. Results

### 3.1. Clinical Characteristics of the Study Group

A total of 46 eyes from 44 patients with FTMHs were included in the study. All patients underwent pars plana vitrectomy with the inverted ILM flap technique at the 1st Department of Ophthalmology, Pomeranian Medical University in Szczecin, Poland. The study population included 7 men and 37 women, with a mean age of 69.6 years. The detailed baseline characteristics are presented in Table 1.

### 3.2. Temporal Dynamics of Retinal Remodelling After Surgery

#### 3.2.1. Anatomical Closure and Integrity of the Outer Retinal Layer

Successful anatomical closure was achieved in all eyes (100%) throughout the 12-month follow-up period. Restoration of the outer retinal layers demonstrated a characteristic sequential pattern (Table 2). At 1 month postoperatively, integrity of the ELM was observed in 23 eyes (50.0%), whereas complete restoration of the EZ was present in only 5 eyes (10.9%). Between 1 and 6 months, both parameters improved significantly, with ELM integrity increasing to 73.9% (*p* = 0.003) and EZ integrity increasing to 50.0% (*p* < 0.001). Only minor additional improvement was observed between 6 and 12 months, reaching 78.3% and 56.5%, respectively (all *p* > 0.05).

#### 3.2.2. Evolution of the Closure Pattern

Structural changes were reflected by progressive improvement in the overall closure pattern (Table 2). Complete restoration of both the ELM and the EZ (Type 1) was observed in only 8.7% of eyes at 1 month, but increased to 45.7% at 6 months and became the predominant postoperative configuration at 12 months (54.3%). Conversely, the proportion of eyes with persistent disruption of both outer retinal layers (Type 3) decreased from 43.5% at 1 month to 23.9% at 6 months, with a further modest reduction to 21.7% by 12 months (*p* = 0.04). Overall, the most pronounced improvement in the retinal microstructure occurred between 1 and 6 months after surgery, although the distribution of the closure pattern continued to shift to a lesser extent thereafter.

#### 3.2.3. Evolution of Glial Proliferation

Postoperative glial proliferation demonstrated a similar temporal pattern (Table 2). The severity of gliosis decreased significantly between 1 and 6 months (*p* = 0.004), whereas no further significant changes were observed between 6 and 12 months (*p* = 0.18). The prevalence of Grade 3 glial proliferation decreased from 30.4% to 15.2% at 6 months, whereas Grade 1 glial proliferation became the predominant postoperative phenotype (45.7%) from the sixth postoperative month onwards. Likewise, macular volume progressively decreased throughout the follow-up period, with significant reductions observed between all the study visits (all *p* < 0.001). As complementary structural information, longitudinal retinal thickness measurements across all nine ETDRS subfields at 1, 6, and 12 months postoperatively are presented in Supplementary Table S1.

Trajectory analysis further demonstrated that postoperative glial remodelling was characterised predominantly by regression rather than progression. Half of the eyes with Grade 3 proliferation remained unchanged during follow-up, whereas the remaining cases gradually regressed to lower grades. A similar tendency was observed in Grade 2 proliferation, with half of the eyes regressing to Grade 1 by 6 months. In contrast, superficial gliosis (Grade 1) proved remarkably stable, remaining unchanged in 92.9% of the eyes throughout the observation period (Table 3). Interobserver agreement for OCT-based morphological grading was high, with weighted Cohen’s κ values of 0.934 for glial proliferation grading and 0.967 for closure-pattern classification.

#### 3.2.4. Correlation Between Glial Proliferation and Retinal Structure

The degree of postoperative glial proliferation consistently reflected the extent of structural retinal restoration. Significant positive correlations were observed between the glial proliferation grade and the closure pattern at all postoperative visits (Rs = +0.685 at 1 month, Rs = +0.633 at 6 months, and Rs = +0.570 at 12 months; all *p* < 0.001), indicating that more extensive outer retinal disruption was associated with greater postoperative gliosis.

### 3.3. The Functional Recovery After Surgery

#### 3.3.1. Visual Acuity and Retinal Sensitivity

BCVA improved progressively throughout the entire follow-up period (Table 4). Average retinal sensitivity significantly increased between 1 and 6 months postoperatively (*p* < 0.001), followed by stabilisation between 6 and 12 months (*p* = 0.09), indicating that most improvement in retinal sensitivity occurred during the early postoperative period. Significant positive correlations between retinal sensitivity and visual acuity were observed at all postoperative visits (Rs = +0.416 (*p* = 0.004) at 1 month; Rs = +0.524 (*p* < 0.001) at 6 months and Rs = +0.653 (*p* < 0.001) at 12 months.

Notably, median BCVA increased from 48 ETDRS letters preoperatively to 67 letters at 12 months, corresponding to a 19-letter difference in group medians. The largest increase in median BCVA was observed between baseline and 1 month, from 48 to 59 letters, followed by a further gradual increase to 67 letters at 12 months. The proportion of eyes with average retinal sensitivity within the reference range increased from 39.1% (18/46) at 1 month to 65.2% (30/46) at 6 months and 67.4% (31/46) at 12 months.

#### 3.3.2. Fixation Stability Analysis

Fixation stability followed a temporal pattern similar to that of retinal sensitivity. The most pronounced improvement occurred between 1 and 6 months postoperatively, after which the fixation parameters remained largely stable.

The mean fixation stability P1 increased from 91.22 ± 11.44% at 1 month to 94.07 ± 8.07% at 6 months (*p* = 0.003), with no significant further improvement by 12 months (*p* = 0.18). P2 values remained stable throughout follow-up (all *p* > 0.05).

Analysis of the BCEA demonstrated a reduction in fixation area between 1 and 6 months postoperatively. The mean 95% BCEA areas decreased from 3.30 ± 3.30 deg^2^ to 2.43 ± 2.28 deg^2^ (*p* = 0.01), primarily reflecting contraction of the horizontal semiaxis (*p* = 0.03), whereas the vertical parameters remained unchanged. Similar findings were observed for the 63% BCEA, with a significant reduction in the area between 1 and 6 months (*p* = 0.01), followed by their stabilisation thereafter.

#### 3.3.3. Electrophysiological Recovery

Electrophysiological improvement was confined to the central foveal region. The mean mfERG P1-wave amplitude density in Ring 1 significantly increased from 66.95 ± 28.29 nV/deg^2^ at 1 month to 78.52 ± 31.80 nV/deg^2^ at 6 months (*p* = 0.03), followed by a non-significant trend towards further improvement at 12 months (*p* = 0.09). Similarly, the P1-wave implicit time in Ring 1 remained stable throughout the study period. The proportion of eyes with mfERG P1 response density in R1 within the reference range increased from 47.8% (22/46) to 67.4% (31/46) and 73.9% (34/46), respectively. No consistent longitudinal pattern was observed in the more peripheral rings (R2–R6). P1 response density remained statistically unchanged across these rings, whereas isolated significant differences in P1 implicit time were observed without a consistent spatial or temporal pattern (Supplementary Table S2).

### 3.4. Structure–Function Relationships During Postoperative Retinal Healing

#### 3.4.1. Closure Pattern and Visual Recovery

**Figure 3:**
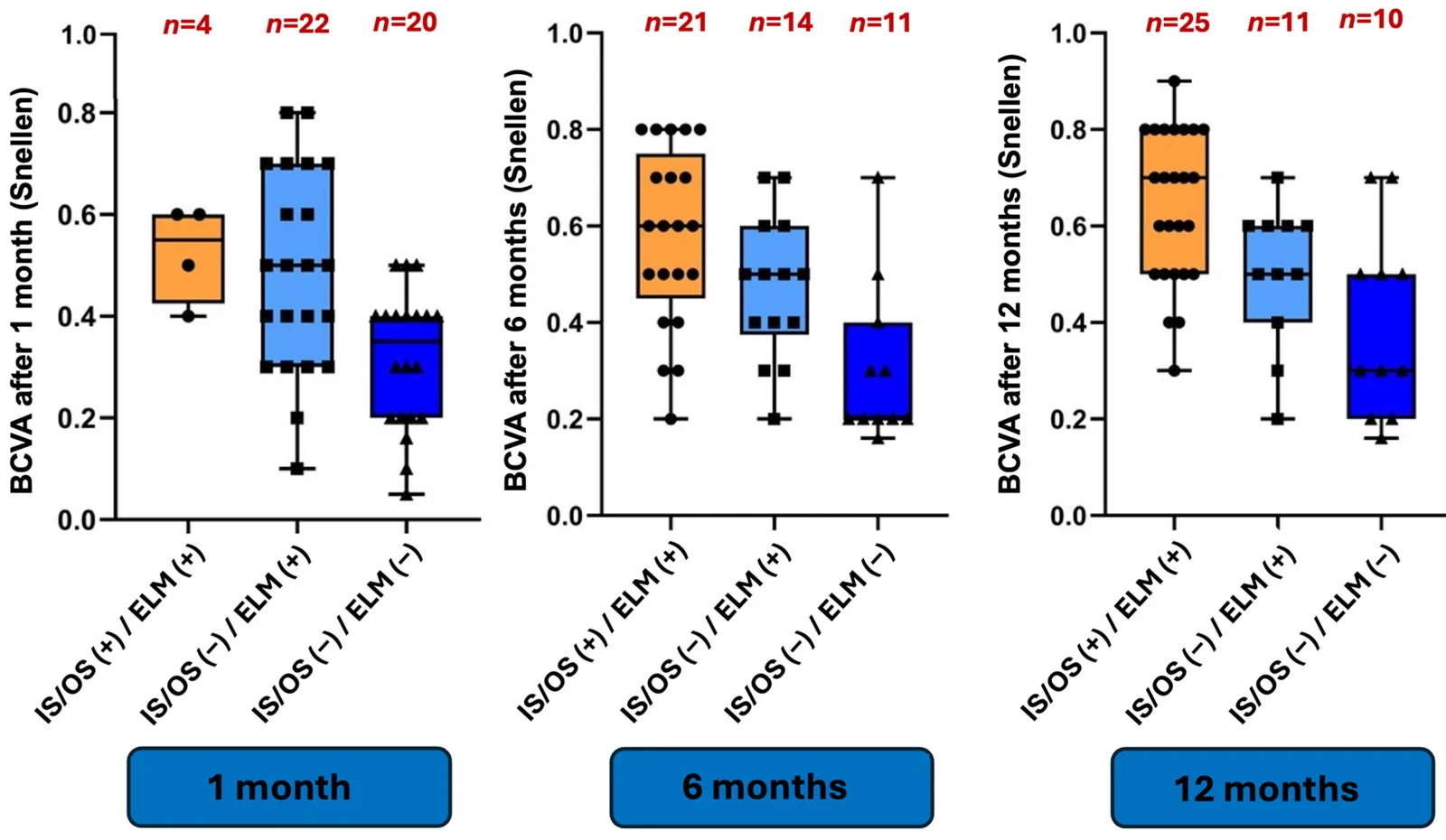
Associations between Postoperative Retinal Morphology (Closure Pattern) and Best-Corrected Visual Acuity at 1, 6, and 12 Months After Surgery.

The recovery pattern of the outer macular layers demonstrated consistent associations with postoperative visual function at all follow-up visits. Moderate negative correlations between closure pattern and BCVA, expressed in Snellen decimal notation for the correlation analyses, were observed at 1 month (Rs = −0.480; *p* < 0.001), 6 months (Rs = −0.532; *p* < 0.001), and 12 months (Rs = −0.556; *p* < 0.001). Thus, eyes with more complete restoration of the outer retinal layers consistently achieved better postoperative visual acuity. The data in Figure 3 further demonstrate a stepwise decline in postoperative visual acuity with increasing closure-pattern type, with eyes exhibiting Type 1 closure consistently achieving the highest median BCVA, followed by those with Type 2 and Type 3 closure patterns at all postoperative visits.

A different pattern was observed for retinal sensitivity. The correlation with the closure pattern was weak and not statistically significant at 1 month (Rs = −0.146; *p* = 0.33), but was significant at 6 months (Rs = −0.396; *p* = 0.006) and 12 months (Rs = −0.405; *p* = 0.005).

At 12 months, closure pattern was negatively correlated with mfERG R1 P1 response density (Rs = −0.328; *p* ≤ 0.05).

#### 3.4.2. Glial Proliferation and Visual Recovery

Postoperative glial proliferation demonstrated consistent associations with visual function throughout follow-up. Increasing gliosis severity was consistently correlated with poorer visual acuity throughout follow-up (Rs = −0.648 at 1 month, Rs = −0.654 at 6 months, and Rs = −0.657 at 12 months; all *p* < 0.001), indicating that more extensive postoperative gliosis was associated with less favourable visual recovery.

Glial proliferation was also associated with retinal sensitivity. The correlation was weak at 1 month (Rs = −0.288; *p* = 0.05), whereas significant moderate negative correlations were observed at 6 months (Rs = −0.493; *p* < 0.001) and 12 months (Rs = −0.547; *p* < 0.001).

More extensive glial proliferation was additionally associated with poorer fixation stability (at 12 months, Rs = −0.367; *p* ≤ 0.05), particularly for the 63% horizontal BCEA parameters (Rs = 0.377; *p* ≤ 0.05).

Importantly, in an exploratory multivariable regression model lower glial proliferation grade (B = −0.091, 95% CI: −0.155 to −0.027; *p* = 0.006), lower closure-pattern grade (B = −0.073, 95% CI: −0.141 to −0.005; *p* = 0.035), and better preoperative BCVA (B = 0.564, 95% CI: 0.036 to 1.092; *p* = 0.037) were independently associated with better BCVA at 12 months after adjustment for age and minimum linear hole diameter. Given the absence of adjustment for multiple comparisons, the structure–function associations should be interpreted as exploratory.

## 4. Discussion

The present study shows that retinal healing after inverted ILM flap surgery follows a time-dependent course involving both structural and functional changes rather than representing a single postoperative event. By combining serial OCT assessment with multimodal functional testing, we showed that regression of postoperative glial proliferation, restoration of the outer retinal architecture, and recovery of retinal function occurred in parallel, with the greatest changes occurring between 1 and 6 months postoperatively. Although structural and functional recovery largely stabilised thereafter, significant associations between retinal morphology and visual function were observed throughout postoperative follow-up.

The concept of surgical success in macular hole surgery has evolved substantially over the past decade. While anatomical closure was previously regarded as the principal outcome, the consistently high closure rates achieved with the inverted ILM flap technique have shifted attention towards the quality of postoperative retinal restoration and its relationship with visual recovery. Previous OCT studies have demonstrated that reconstruction of the outer retina follows a sequential process, with restoration of the ELM preceding recovery of the EZ [11,12,17]. Carpineto et al. [21] further showed that the most pronounced structural remodelling occurs during the early postoperative period. Our results are consistent with these observations and additionally indicate that this structural recovery closely parallels functional improvement. Furthermore, the proportion of eyes achieving complete restoration of both the ELM and the EZ at 12 months (54.3%) is consistent with recent comparative studies [22,23].

Postoperative glial proliferation remains one of the most debated aspects of retinal healing after macular hole surgery [24,25]. Experimental and histopathological studies have indicated that activated Müller cells migrate into foveal defects, where they form a transient scaffold that facilitates tissue approximation and outer retinal reconstruction [26,27,28]. However, excessive or persistent gliosis has been associated with incomplete restoration of the outer retinal layers and poorer visual outcomes. Iwasaki et al. [13] demonstrated that greater postoperative glial tissue is associated with impaired restoration of the ONL, ELM and EZ, accompanied by worse visual acuity, whereas Qi et al. [10] emphasised that postoperative gliosis is a dynamic process rather than a static OCT finding. Our findings are consistent with these observations, showing gradual regression of glial proliferation over time alongside improvements in retinal sensitivity, fixation stability, and electrophysiological function. This parallel temporal pattern indicates an association between structural remodelling and functional improvement.

Functional recovery following macular hole surgery extends beyond visual acuity alone. Previous studies have shown that retinal sensitivity assessed by microperimetry improves after successful surgery and is correlated with the restoration of the outer retinal layers, indicating that microperimetry captures functional changes that are not fully reflected by conventional visual acuity measurements [21,29,30]. Although mfERG has been incorporated into relatively few studies of postoperative macular hole recovery [31,32], it provides an objective assessment of central retinal function that complements OCT and psychophysical testing [33]. In the present study, retinal sensitivity, fixation stability, and central mfERG responses all showed their greatest improvement between the 1- and 6-month postoperative assessments, closely paralleling structural retinal remodelling. These exploratory findings indicate that different functional modalities reflect complementary aspects of postoperative retinal recovery. Whereas microperimetry primarily characterises localised retinal sensitivity and fixation behaviour, mfERG provides objective evidence of electrophysiological recovery of the central retina. Unlike previous studies focusing on outer retinal restoration [12], microperimetric outcomes [14,15,21], or mfERG changes [31,32], our study integrates these structural and functional parameters together with postoperative glial remodelling, allowing the temporal interrelationships of these parameters during retinal healing to be evaluated within the same cohort.

An important finding of the present study is that structure–function associations are observed throughout postoperative follow-up. Previous studies have shown that the restoration of the ELM and EZ, as well as post-operative foveal glial tissue, is associated with visual outcome after macular hole surgery [2,3,12,13,34]. Although several longitudinal studies have described the temporal course of structural and functional recovery [2,4,8,12,34], the longitudinal relationship between postoperative retinal morphology and functional outcomes remains less well characterised. In the present study, significant associations between retinal morphology and functional outcomes were observed throughout postoperative follow-up. Importantly, exploratory multivariable analysis further showed that lower glial proliferation grade, lower closure-pattern grade, and better preoperative BCVA were independently associated with better BCVA at 12 months after adjustment for age and minimum linear hole diameter. These exploratory findings indicate that both postoperative glial proliferation and outer retinal restoration are independently associated with long-term visual outcome. This pattern may reflect the gradual resolution of transient postoperative changes, including photoreceptor reorganisation and glial remodelling, which may alter the relationship between retinal morphology and visual function as postoperative recovery progresses [8,13,27,35]. Consequently, OCT biomarkers obtained during the early postoperative period should be interpreted cautiously, as their functional significance may evolve during ongoing retinal remodelling.

Postoperative structural and functional findings should also be interpreted in the context of baseline macular hole characteristics. In our previous study [20], smaller minimum macular hole diameter and shorter symptom duration were independently associated with a more favourable long-term visual outcome, highlighting the relevance of baseline anatomical characteristics and disease duration to postoperative interpretation. The present study complements these findings by focusing on the longitudinal evolution of postoperative glial proliferation, closure pattern, outer retinal restoration, and functional outcomes. Notably, baseline functional status also remained relevant in the current exploratory multivariable analysis, in which better preoperative BCVA was independently associated with better BCVA at 12 months after adjustment for age, minimum linear hole diameter, closure pattern, and glial proliferation grade. Accordingly, postoperative morphological and functional findings should be interpreted within the broader context of the preoperative characteristics of the treated eyes.

From a clinical perspective, median ETDRS BCVA increased from 48 letters preoperatively to 67 letters at 12 months, corresponding to a 19-letter difference in group medians. For retinal sensitivity, BCEA, and mfERG, minimum clinically important differences specifically applicable to postoperative FTMH are not well established. As complementary clinical context, the proportion of eyes reaching the reference range for retinal sensitivity increased from 39.1% at 1 month to 67.4% at 12 months, while the corresponding proportion for mfERG R1 P1 response density increased from 47.8% to 73.9%. Although reaching the reference range should not be interpreted as an established threshold for clinically meaningful improvement, these findings may help inform patients that functional recovery can continue beyond anatomical hole closure and the early postoperative period.

These findings also indicate that postoperative assessment after inverted ILM flap surgery should extend beyond the confirmation of anatomical hole closure. Although successful closure remains essential, it does not fully capture the biological processes underlying the restoration of retinal function. Given that the most pronounced structural and functional changes were observed between 1 and 6 months, this interval appears particularly informative for postoperative monitoring. Integration of OCT with complementary functional techniques, such as microperimetry, fixation analysis, and mfERG, may provide a more comprehensive assessment of retinal healing and help identify eyes with delayed functional recovery despite successful anatomical closure.

Taken together, our findings integrate serial OCT biomarkers, postoperative glial remodelling, and multimodal functional assessment to provide a longitudinal perspective on retinal healing following inverted ILM flap surgery.

### Limitations

This study has several limitations. First, its retrospective design and relatively small sample size may limit the generalizability of the findings. Furthermore, the inclusion of only eyes with complete postoperative assessments at 1, 6, and 12 months may have introduced selection and loss-to-follow-up bias, potentially favouring patients with more complete follow-up and reducing the representativeness of the study cohort. Importantly, the 100% anatomical closure rate observed in this study pertains to this selected cohort of eyes with complete 12-month follow-up and should not be interpreted as an estimate of the overall effectiveness of the inverted ILM flap technique in an unselected surgical population. In addition, the absence of a control group limits direct comparison with alternative surgical approaches. Second, OCT-based morphological grading was performed primarily by a single experienced retinal specialist, which may introduce a degree of subjectivity in the classification of closure patterns and glial proliferation. Furthermore, given the limited sample size, the multivariable analyses should be considered exploratory, and no formal adjustment for multiple comparisons was applied. Therefore, the exploratory structure–function associations may be susceptible to type I error and should be interpreted cautiously. In addition, two patients contributed both eyes, which were treated as independent observations without adjustment for within-subject correlation; this should be considered when interpreting the exploratory statistical findings. Finally, the study was conducted at a single center, and the findings should therefore be interpreted within the context of the study population and design. The present analysis focused specifically on longitudinal postoperative changes, while the predictive value of preoperative anatomical and functional parameters has been evaluated separately in our previous study [20]. Nevertheless, the longitudinal assessment of structural, functional, and electrophysiological parameters over a 12-month follow-up provides a comprehensive multimodal characterization of postoperative retinal recovery.

## 5. Conclusions

In conclusion, the results of the present study indicate that retinal healing after inverted ILM flap surgery follows a time-dependent course involving both structural andfunctional changes rather than representing a single anatomical event. Regression of postoperative glial proliferation, restoration of the outer retinal microstructure, and recovery of retinal function occur in parallel, predominantly between 1 and 6 months postoperatively, while exploratory associations between retinal morphology and functional outcomes persist throughout the healing process. The observed temporal pattern of postoperative gliosis, with more extensive proliferation at earlier postoperative stages followed by gradual regression alongside outer retinal restoration and functional improvement, is compatible with a reparative–remodelling process, although its biological role cannot be directly established from the present observational data. Longitudinal integration of OCT, microperimetry, fixation analysis, and mfERG provides a comprehensive framework for understanding retinal healing after macular hole surgery and may improve postoperative evaluation.

## Figures and Tables

**Table 1:** Baseline demographic, anatomical and functional characteristics of the study cohort.

Parameter	Value
Age	69.6 ± 5.5
Gender (F/M)	37/7
Duration of disease, [*n*] (%)	
<6 M	22 (47.8%)
6–12 M	16 (34.8%)
>12 M	8 (17.4%)
FTMH size Category (CLOSE classification)	
Small (≤250 µm)	2 (4.3%)
Medium (250–400 µm)	11 (23.9%)
Large (400–550 µm)	19 (41.3%)
XL (550–800 µm)	14 (30.4%)
BCVA [Snellen charts] Median (IQR)	0.16 (0.15)
BCVA [ETDRS charts] Median (IQR)	48 (4)
Minimum linear hole diameter (µm) mean ± SD	469.1 ± 149.6
Basal hole diameter (BD, µm) mean ± SD	1048.2 ± 261.6
Average retinal sensitivity [dB], median (IQR)	22.35 (4.1)
Fixation Stability P1 [%], median (IQR)	89 (28)
Fixation Stability P2 [%], median (IQR)	99.5 (4)
63% BCEA: area [deg^2^], median (IQR)	1.3 (2.6)
95% BCEA: area [deg^2^], median (IQR)	4 (7.7)
mfERG R1 P1-wave amplitude [nV/deg^2^], median (IQR)	40.69 (38.39)
mfERG R1 P1-wave implicit time [ms], median (IQR)	44.605 (5.8)

Data are presented as the mean ± standard deviation (SD) for normally distributed variables and median (interquartile range, IQR) for non-normally distributed variables.

**Table 2:** Postoperative evolution of OCT-based foveal structural changes during the 12-month follow-up.

Parameter	1 Month	6 Month	12 Month	*p* (1 vs. 6)	*p* (1 vs. 12)	*p* (6 vs. 12)
EZ integrity present [*n*] (%)	5 (10.9%)	23 (50%)	26 (56.5%)	<0.001 ^a^	<0.001 ^a^	0.25 ^a^
ELM integrity present [*n*] (%)	23 (50%)	34 (73.9%)	36 (78.3%)	0.003 ^a^	<0.001 ^a^	0.48 ^a^
The closure pattern [*n*] (%)						
1	4 (8.7%)	21 (45.7%)	25 (54.3%)	<0.001 ^b^	<0.001 ^b^	0.04 ^b^
2	22 (47.8%)	14 (30.4%)	11 (23.9%)			
3	20 (43.5%)	11 (23.9%)	10 (21.7%)			
Glial proliferation grade [*n*] (%)						
0	8 (17.4%)	8 (17.4%)	9 (19.6%)	0.004 ^b^	0.003 ^b^	0.18 ^b^
1	14 (30.4%)	21 (45.7%)	21 (45.7%)			
2	10 (21.7%)	10 (21.7%)	9 (19.6%)			
3	14 (30.4%)	7 (15.2%)	7 (15.2%)			
Macular Volume—median (IQR) [mm^3^]	8.86 (0.6)	8.585 (0.68)	8.555 (0.74)	<0.001 ^b^	<0.001 ^b^	<0.001 ^b^

Data are presented as *n* (%) or median (IQR). Closure pattern classification: type 1—complete restoration of both the EZ and ELM; type 2—disrupted EZ with intact ELM; type 3—disruption of both the EZ and ELM. Glial proliferation grading: grade 0—no glial proliferation; grade 1—superficial proliferation above the ELM; grade 2—proliferation at and above the ELM; grade 3—proliferation extending through all retinal layers. ^a^ McNemar’s test, ^b^ Wilcoxon signed-rank test.

**Table 3:** Evolution of different glial proliferation types during the 12-month follow-up period.

Type of Glial Proliferation			Eyes, *n* (%)
1 Month	6 Months	12 Months	
Grade 3 (*n* = 14)	3	3	7 (50.0)
	2	2	4 (28.6)
		1	1 (7.1)
	1	1	2 (14.3)
Grade 2 (*n* = 10)	2	2	5 (50.0)
	1	1	4 (40.0)
		0	1 (10.0)
Grade 1 (*n* = 14)	1	1	13 (92.9)
	0	0	1 (7.1)
Grade 0 (*n* = 8)	0	0	7 (87.5)
	1	1	1 (12.5)

**Table 4:** Postoperative functional changes during the 12-month follow-up.

Parameter	1 Month Median (IQR) Mean ± SD	6 Month Median (IQR) Mean ± SD	12 Month Median (IQR) Mean ± SD	*p* * 1 vs. 6	*p* * 6 vs. 12
BCVA (Snellen)	0.4 (0.2) 0.415 ± 0.185	0.5 (0.3) 0.486 ± 0.199	0.55 (0.3) 0.543 ± 0.2	**0.006**	**<0.001**
BCVA (ETDRS)	59 (14) 59.96 ± 9.28	63.5 (16) 62.41 ± 8.82	67 (11) 65.435 ± 9.30	**0.002**	**<0.001**
Average retinal sensitivity [dB]	24.3 (2.7) 23.86 ± 2.41	25.4 (1.8) 25.07 ± 2.77	25.55 (2.5) 25.51 ± 1.95	**<0.001**	0.09
Fixation Stability P1 [%]	96.5 (7) 91.22 ± 11.44	97 (5) 94.07 ± 8.07	97.5 (6) 94.80 ± 7.88	**0.003**	0.18
Fixation Stability P2 [%]	100 (1) 98.89 ± 2.24	100 (1) 99.24 ± 1.51	100 (0) 99.46 ± 1.21	0.27	0.39
63% BCEA: horizontal [°]	1 (0.5) 1.12 ± 0.53	0.9 (0.4) 0.97 ± 0.42	0.9 (0.5) 0.98 ± 0.40	**0.02**	0.86
63% BCEA: vertical [°]	0.9 (0.9) 1.07 ± 0.62	0.85 (0.7) 0.93 ±0.51	0.7 (0.6) 0.85 ±0.49	0.06	0.19
63% BCEA: area [deg^2^]	0.6 (1.1) 1.09 ± 1.10	0.6 (0.7) 0.81 ± 0.76	0.5 (0.7) 0.73 ± 0.64	**0.01**	0.28
95% BCEA: horizontal [°]	1.75 (0.9) 1.95 ± 0.91	1.6 (0.7) 1.68 ± 0.72	1.5 (0.7) 1.67 ± 0.69	**0.03**	0.84
95% BCEA: vertical [°]	1.5 (1.5) 1.84 ± 1.10	1.5 (1.2) 1.61 ± 0.89	1.2 (0.9) 1.47 ± 0.84	0.06	0.17
95% BCEA: area [deg^2^]	1.85 (3.2) 3.30 ± 3.30	1.75 (1.9) 2.43 ± 2.28	1.4 (2) 2.15 ± 1.91	**0.01**	0.18
mfERG R1 P1-wave amplitude [nV/deg^2^]	60.05 (42.4) 66.95 ± 28.29	73.85 (32.21) 78.52 ± 31.80	81.83 (43.33) 86.97 ± 33.12	**0.03**	0.09
mfERG R1 P1-wave implicit time [ms]	46.6 (4.9) 46.71 ± 3.78	47.1 (3.9) 46.4 ± 4.21	46.1 (3.9) 46.23 ± 3.51	0.92	0.69

* Wilcoxon signed-rank test.

## Data Availability

The data that were used to support the findings of this study are available from the corresponding author upon request.

## Supplementary Materials

The following supporting information can be downloaded at: https://www.mdpi.com/article/10.3390/diagnostics16183017/s1, Table S1: Longitudinal retinal thickness measurements across ETDRS subfields during postoperative follow-up; Table S2: Longitudinal mfERG parameters across retinal rings R1–R6.
