# Supplementary material for: Long-Term Structural and Functional Restoration of the Retina After Inverted Internal Limiting Membrane Flap Surgery for Idiopathic Full-Thickness Macular Holes
Source: Diagnostics (Basel). 2026 Sep 17;16(18):3017. doi: 10.3390/diagnostics16183017 (PMC13605231; doi:10.3390/diagnostics16183017)
Supplement: Supplementary file 1 [file diagnostics-16-03017-s001.zip › diagnostics-4500031-supplementary.pdf]

**Table S1. Longitudinal retinal thickness measurements across ETDRS subfields during postoperative follow-up**

| Parameter | 1 month Median (IQR) | 6 months Median (IQR) | 12 months Median (IQR) | *p* * 1 vs 6 | *p* * 6 vs 12 |
|---|---|---|---|---|---|
| Central 1 mm | 282.5 (53) | 289 (40) | 295 (36) | 0.08 | 0.28 |
| Inner superior (1-3 mm) | 341 (26) | 333 (30) | 332.5 (26) | **<0.001** | 0.06 |
| Inner inferior (1-3 mm) | 344 (24) | 339.5 (31) | 342 (24) | **<0.001** | 0.17 |
| Inner nasal (1-3 mm) | 358.5 (29) | 355 (33) | 356.5 (30) | 0.17 | 0.33 |
| Inner temporal (1-3 mm) | 344 (24) | 339.5 (31) | 342 (24) | **<0.001** | **0.01** |
| Outer superior (3-6 mm) | 309 (21) | 298 (30) | 293.5 (25) | **<0.001** | **<0.001** |
| Outer inferior (3-6 mm) | 302 (21) | 292 (30) | 292 (27) | **<0.001** | **0.004** |
| Outer nasal (3-6 mm) | 325 (27) | 314 (27) | 314 (24) | **<0.001** | 0.1 |
| Outer temporal (3-6 mm) | 291.5 (23) | 282 (23) | 281 (26) | **<0.001** | **0.003** |

* Wilcoxon signed-rank test

Table S1. Retinal thickness measurements are presented as median (interquartile range [IQR]) in µm at 1, 6, and 12 months postoperatively. Measurements are reported for all nine ETDRS subfields: the central 1-mm subfield, four inner-ring subfields (1–3 mm), and four outer-ring subfields (3–6 mm).

**Table S2. Longitudinal mfERG parameters across retinal rings R1–R6**

| Parameter | 1 month Median (IQR) | 6 months Median (IQR) | 12 months Median (IQR) | *p* * 1 vs 6 | *p* * 6 vs 12 |
|---|---|---|---|---|---|
| R1 P1-wave amplitude [nV/deg$^2$] | 60.05 (42.2) | 73.845 (32.21) | 81.83 (43.33) | **0.03** | 0.09 |
| R2 P1-wave amplitude [nV/deg$^2$] | 46.49 (25.67) | 48.56 (26.41) | 54.385 (22.4) | 0.33 | 0.36 |
| R3 P1-wave amplitude [nV/deg$^2$] | 34.16 (18.89) | 34.785 (16.43) | 34.825 (12.56) | 0.85 | 0.96 |
| R4 P1-wave amplitude [nV/deg$^2$] | 23.25 (12.74) | 23.74 (10.93) | 25.17 (8.9) | 0.67 | 0.41 |
| R5 P1-wave amplitude [nV/deg$^2$] | 17.225 (9.87) | 19.145 (8.47) | 19.89 (9.55) | 0.08 | 0.87 |
| R6 P1-wave amplitude [nV/deg$^2$] | 13.18 (8.56) | 15.255 (7.76) | 14.915 (7.6) | 0.38 | 0.8 |
| R1 P1-wave implicit time [ms] | 46.6 (4.9) | 47.1 (3.9) | 46.1 (3.9) | 0.92 | 0.69 |
| R2 P1-wave implicit time [ms] | 44.1 (3.9) | 43.1 (2.9) | 42.2 (1.9) | 0.78 | **0.03** |
| R3 P1-wave implicit time [ms] | 43.1 (2.9) | 42.2 (2.9) | 42.2 (2.9) | **0.02** | 0.71 |

| | | | | | |
|---|---|---|---|---|---|
| R4 P1-wave implicit time [ms] | 42.2 (1.9) | 42.2 (1.9) | 42.2 (1.9) | 0.07 | 0.86 |
| R5 P1-wave implicit time [ms] | 42.2 (0.9) | 42.2 (1.9) | 42.2 (1.9) | **0.01** | 0.46 |
| R6 P1-wave implicit time [ms] | 42.2 (2.9) | 42.2 (1.9) | 42.2 (1.9) | 0.07 | 0.50 |

* Wilcoxon signed-rank test. Statistically significant *p*-values ($p < 0.05$) are shown in bold. No formal adjustment for multiple comparisons was applied; these secondary regional analyses should therefore be considered exploratory. IQR, interquartile range; mfERG, multifocal electroretinography.
